# Treatment of Central Serous Chorioretinopathy With Topical Difluprednate: A Literature Review

**DOI:** 10.7759/cureus.105298

**Published:** 2026-03-16

**Authors:** Sayan K Chatterjee, Nausheen Khuddus

**Affiliations:** 1 Medicine, University of Central Florida College of Medicine, Orlando, USA; 2 Medicine, HCA Florida North Florida Hospital, Gainesville, USA; 3 Ophthalmology, HCA Florida North Florida Hospital, Gainesville, USA; 4 Ophthalmology, University of Central Florida College of Medicine, Orlando, USA

**Keywords:** bilateral central serous chorioretinopathy, central serous chorioretinopathy, central serous chorioretinopathy (cscr), central serous choroid retinopathy, central serous retinopathy, chronic central serous chorioretinopathy, difluprednate, peripapillary pachychoroid syndrome, topical steroid, undifferentiated pachychoroid spectrum

## Abstract

Central serous chorioretinopathy (CSR) is an eye condition where fluid leakage from the choroid causes a detachment of the macula, the central part of the retina responsible for clear vision. Although the existing literature identifies corticosteroids as a risk factor for CSR, there is a growing interest in the use and impact of corticosteroids to treat CSR. The objective of this review is to evaluate the current evidence on the use of difluprednate, a topical ophthalmic medicine to treat inflammation and pain, for the treatment of CSR.

The PubMed and Google Scholar databases were searched for all relevant English-language studies published from inception to October 2025, using the keywords "central serous chorioretinopathy" and "difluprednate".

Despite the common consensus that steroids contribute to CSR development, many patients continue to be prescribed difluprednate in clinical practice after their CSR diagnosis. The short- and long-term effects of difluprednate for CSR treatment are not well-established. Future clinical trials can provide further insights into the rationale of difluprednate therapy for CSR treatment, especially because its use is contraindicated by existing evidence.

## Introduction and background

Central serous chorioretinopathy (CSR), the most common variant of the pachychoroid disease spectrum (PDS), is characterized by the accumulation of central subretinal fluid (SRF) and localized serous neuroretinal and pigment epithelial detachments (PEDs) [1]. For patients with chronic or recurrent CSR, there could be a risk of permanent vision loss due to progressive degeneration of the retinal pigment epithelium (RPE) [2]. The prevalence of CSR is higher in men than in women in clinic-based populations, though older women showed a similar prevalence as men [3]. Limited evidence from the literature suggests comparable rates of CSR across White, Asian, and African American populations [4].

The pathophysiology of CSR involves two primary mechanisms: choroidal hyperpermeability with dilation and increased vascular permeability of choroidal vessels, and RPE dysfunction that results in focal or multifocal breakdown of the outer blood-retinal barrier, allowing for subretinal fluid accumulation. Corticosteroids are strongly implicated in CSR development through both glucocorticoid receptor (GR) and mineralocorticoid receptor (MR) pathways. Glucocorticoid activation affects choroidal blood flow regulation and increases vascular permeability through effects on tight junction proteins and vascular endothelial growth factor (VEGF) expression. Additionally, glucocorticoids can impair RPE pump function and reduce the phagocytic capacity of RPE cells, compromising their ability to maintain retinal homeostasis. The MR pathway appears particularly relevant to CSR pathogenesis. MR activation in the choroid and RPE leads to increased vascular permeability, altered ion transport, and extracellular fluid accumulation. Many glucocorticoids, including cortisol and synthetic analogues, can activate MRs at physiologic concentrations, especially when 11β-hydroxysteroid dehydrogenase type 2 (11β-HSD2), which normally protects MRs from glucocorticoid activation, is overwhelmed or bypassed.

If corticosteroid signaling exerts context-dependent effects in CSR, it is conceivable that distinct imaging phenotypes may reflect underlying biological heterogeneity. However, no validated multimodal imaging criteria currently define an "inflammatory" CSR subtype. On optical coherence tomography (OCT), classic CSR typically demonstrates serous neurosensory detachment, pachychoroid features with increased choroidal thickness, dilated Haller's layer vessels, and focal RPE alterations. In contrast, findings such as pronounced intraretinal hyperreflective foci, subretinal fibrin, or atypical choroidal thickening patterns have been hypothesized to reflect greater inflammatory or exudative activity, although these features are not specific and may overlap with other entities. Fluorescein angiography (FA) in CSR commonly reveals focal "smokestack" or "inkblot" leakage patterns, whereas diffuse leakage or multifocal staining patterns may suggest alternative diagnoses or broader pachychoroid spectrum involvement. Indocyanine green angiography (ICGA) typically demonstrates choroidal hyperpermeability but does not reliably distinguish inflammatory from non-inflammatory mechanisms. Enhanced depth imaging OCT and swept-source OCT have further characterized pachychoroid morphology; however, no imaging parameter has been validated to predict corticosteroid responsiveness or to identify a steroid-modifiable CSR phenotype. Therefore, any attempt to define an imaging-based subgroup that might theoretically respond differently to corticosteroid modulation remains speculative and warrants prospective investigation.

Treatment options for CSR include conventional laser, half-dose or half-fluence photodynamic therapy (PDT), antioxidants, mineralocorticoid receptor antagonists (MRAs), rifampicin, selective retina therapy (SRT), and subthreshold micropulse laser [5, 6]. Based on the theory that choroidal hyperpermeability is associated with increased VEGF expression, anti-angiogenic or anti-vascular endothelial growth factor (anti-VEGF) drugs have also been advocated for in treating CSR [7].

PDT remains the current gold standard treatment for chronic or recurrent CSR, particularly when utilizing refined half-dose or half-fluence protocols; these modified approaches have significantly improved the safety profile of PDT while maintaining therapeutic efficacy. Half-dose and half-fluence PDT have demonstrated effective closure of choroidal hyperpermeability with a reduced risk of complications when compared to standard-fluence protocols. Emerging CSR treatments also include selective retina therapy (SRT) and photobiomodulation (PBM). SRT, a laser-based treatment that selectively targets RPE cells while sparing the photoreceptors and surrounding neurosensory retina, may stimulate RPE regeneration and restoration of barrier function without causing thermal collateral damage or visible scarring. The proposed mechanisms of PBM (also called low-level light therapy) are improved mitochondrial function and cellular energy production, enhanced blood flow in the choroid, reduced inflammation, and stimulation of RPE function. However, evidence for the efficacy of these therapies is still evolving; they do not serve as standards of practice yet. Although logistical constraints, including verteporfin availability, may affect PDT's access in certain regions, these factors do not diminish the established role of PDT as the current standard of care. Any exploration of alternative therapeutic mechanisms should therefore be viewed as complementary and investigational rather than substitutive.

Comprehensive management of CSR extends beyond ocular interventions to address systemic and lifestyle factors that may contribute to disease development or persistence [1, 6]. The association of obstructive sleep apnea with CSR is explained by intermittent hypoxia leading to choroidal vascular dysregulation and increased sympathetic tone. Stress and psychological factors represent well-recognized contributors to CSR, likely mediated through activation of the hypothalamic-pituitary-adrenal axis and elevated endogenous cortisol levels. Type A personality traits and high-stress occupations are overrepresented in CSR populations. Helicobacter pylori infection has been investigated as a potential systemic risk factor for CSR, with some studies suggesting higher seropositivity rates in CSR patients. The proposed mechanisms include systemic inflammatory responses and effects on cortisol metabolism. However, the evidence for routine H. pylori screening and eradication therapy in CSR management remains inconsistent. Identifying and managing these factors may complement ocular treatments, particularly in recurrent or refractory cases.

Though previous studies found corticosteroid exposure to be a common risk factor for CSR [6, 8-11], two recent studies [12, 13] showed that topical corticosteroids were useful in reducing the intraretinal fluid of patients with a variant of CSR on the spectrum called peripapillary pachychoroid syndrome (PPS), suggesting a more complex and context-dependent role of steroid signaling in choroidal disease. The efficacy of MRAs for CSR treatment remains uncertain, and the drugs may cause side effects, such as gastrointestinal disorders, infection, hyperkalemia, abnormal musculoskeletal and connective tissue, neurological symptoms (intermittent dizziness), and fatigue or a sedative effect [14, 15]. PDT, in addition to its prohibitive cost, is considered an invasive treatment for CSR and may also have adverse effects, such as macular scarring, choroidal neovascularization, and RPE atrophy [14]. In the absence of a single therapy as the standard-of-care, the systemic adverse effects of MRAs, and the risks of treating subfoveal lesions with PDT, managing CSR patients could be a highly individualized process that requires review of different considerations and options [14-16].

Topical corticosteroids are used postoperatively to inhibit the release of arachidonic acid from cell membrane phospholipids, which prevents the formation of leukotrienes and prostaglandins and breaks the inflammatory cascade without the risk of systemic adverse effects. Prednisolone acetate 1%, widely used for many years, controls inflammation effectively, but it has not been shown to consistently address postoperative pain and discomfort. Difluprednate (difluoroprednisolone butyrate acetate, or DFBA) ophthalmic emulsion 0.05% (Durezol®) is a synthetic difluorinated prednisolone derivative originally developed in Japan as a dermatologic ointment. It was the first potent corticosteroid to be approved by the US Food and Drug Administration in 2008 for the treatment of inflammation and pain associated with ocular surgery. Its benefits include its high potency, with the glucocorticoid binding affinity of its active metabolite demonstrated to be 56 times stronger than prednisolone. It also has strong penetrability and a favorable safety profile [17-19]. However, as with all corticosteroids, elevated intraocular pressure (IOP) and acceleration of cataract formation are major concerns with difluprednate. Difluprednate use may also trigger or exacerbate CSR, likely through glucocorticoid receptor-mediated effects on choroidal vasculature and fluid homeostasis. Despite these risks, anecdotal reports continue to document difluprednate use in certain CSR presentations, potentially targeting localized inflammatory components or specific CSR subtypes. Difluprednate matters in inflammation-driven CSR because: 1) in some cases, inflammatory mechanisms have been hypothesized to contribute to choroidal permeability in CSR, though this concept remains speculative and has not been clearly established; 2) steroids remove the dominant pathological driver; and 3) potent posterior penetration is required to affect the choroid and RPE. In cortisol-driven CSR, steroids amplify the disease mechanism, so the same drug flips from therapy to toxin. Considering possible risks and benefits, in many situations, the steroid is typically tapered gradually after its initial introduction and ultimately discontinued to minimize potential adverse effects on CSR.

Not all corticosteroids are pharmacologically equivalent. While several topical corticosteroids are available, difluprednate differs in its fluorinated structure, enhanced lipophilicity, and high intraocular bioavailability, which may allow greater posterior segment penetration compared to agents such as prednisolone acetate or dexamethasone. These pharmacologic characteristics provide a rationale for examining this specific molecule in the context of CSR pathophysiology. Therefore, this narrative literature review focuses specifically on difluprednate to explore whether its unique pharmacologic profile warrants separate consideration in the context of CSR. The intent here is not to advocate for its routine clinical use, but to critically evaluate whether existing evidence supports or refutes its theoretical and observed applications. The research question is broad and exploratory, seeking to analyze relevant theoretical perspectives, outline proposed hypotheses regarding difluprednate use, and examine emerging and evolving concepts within this area.

## Review

Search strategy

A structured literature review was conducted using the PubMed and Google Scholar databases to search for all relevant studies published from inception to November 2025. For PubMed, the medical subject heading keywords used were "central serous chorioretinopathy" and "difluprednate" with the Boolean operator "OR" to retrieve contextual information on each. However, to ensure the precision of the search strategy, the keywords were combined using the Boolean operator "AND". Reference lists in retrieved articles were also reviewed. A regular Google search was undertaken to ensure that the most recent information on the topic, including abstracts and conference presentations, dissertations, and working papers, was not missed. 

Inclusion and exclusion criteria

Only studies published in English were included. Studies that focused on either distantly related or unrelated topics such as retinitis, ocular sarcoidosis, ocular hypotony, pars planitis, CSR, and other eye diseases linked to multiple types of COVID-19 vaccinations were excluded.

Quality assessment

This is a traditional literature review focusing on an exploratory topic of emerging interest. Cochrane's risk of bias tools for randomized trials [20] and non-randomized follow-up studies of exposure effects [21] were earmarked for evaluating quality assessment in case any relevant study for such assessment was identified.

Summary of evidence 

**Figure 1:**
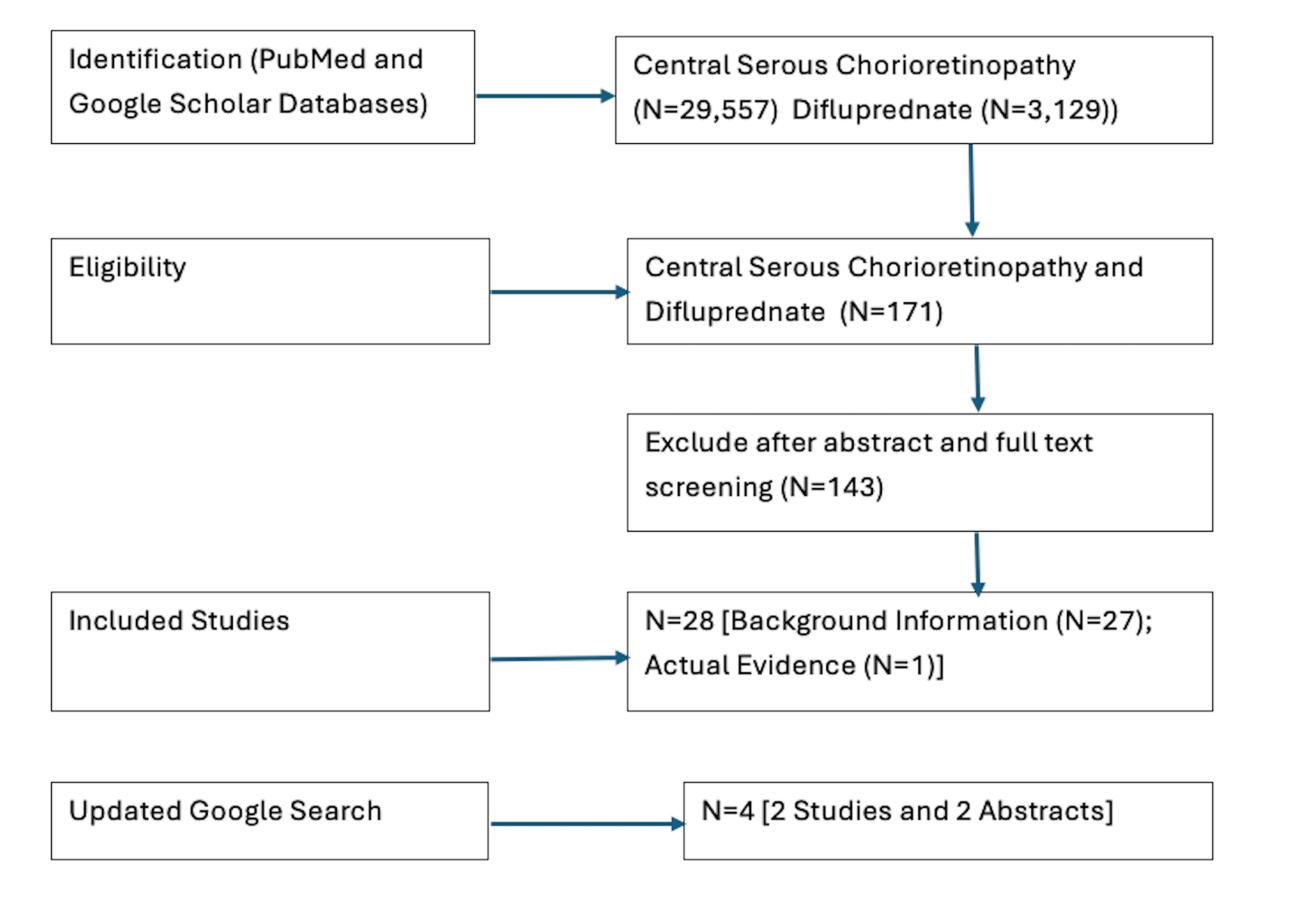
Literature search yield (database inception to November 2025)

The PubMed search with the keywords "CSR" or "difluprednate" yielded 3457 and 189 studies, respectively, while combining the keywords with the Boolean operator "AND" produced only one study [22]. This study found acute exudative maculopathy after half-fluence PDT and/or very minimal fluence PDT for two patients with CSR and age-related macular degeneration (AMD), respectively. Resolution of vision loss occurred after intravitreal bevacizumab treatment for the CSR patient and with topical difluprednate treatment for the AMD patient. A search within the Google Scholar database produced 26,100 and 2940 studies for CSR and difluprednate, respectively, while an exact matching of both CSR and difluprednate yielded 170 studies. After abstract and full text screening, 143 studies were excluded. A regular Google search yielded four more results: two published manuscripts and two abstracts. Figure 1 presents the screening process for included studies in this review. 

Most of the identified studies cite the effectiveness of difluprednate as the mainstay for treating uveitic cystoid macular edema. Uveitis is a general term to describe inflammation of the uvea, the middle layer of the eye. Vogt-Koyanagi-Harada (VKH) disease, a specific case of uveitis, is a systemic autoimmune disorder that affects pigmented tissues, including the eyes, ears, and meninges. For the eyes, it presents as bilateral granulomatous panuveitis, or inflammation of the entire uvea. Unlike VKH, CSR is a vascular disease of the choroid, the layer beneath the retina. Both VKH and severe CSR demonstrate the key symptoms of blurriness and distortion of vision caused by a serous retinal detachment, but CSR does not involve any systemic symptoms, such as prodromal flu-like illness, followed by headaches, dizziness, neck stiffness, and auditory symptoms like tinnitus and hearing loss that are common in cases of VKH disease. Additionally, while some studies documented heightened levels of pro-inflammatory cytokines, such as IL-6, in CSR patients, the connection between CSR and systemic inflammation is still under debate [23]. In some cases, CSR may not demonstrate classic inflammatory signs like pain, redness, heat, or swelling that will automatically trigger the administration of anti-inflammatory steroid drops.

A 2016 study [24] presented the case of a 56-year-old Hispanic man with VKH whose bilateral serous retinal detachments and panuveitis were treated with difluprednate alone. The authors provided the rationale of difluprednate alone as the initial line of treatment because the patient had diabetes, and systemic steroids can cause severe elevation and fluctuation of blood sugar levels. Topical ocular therapy alone also avoids possible side effects associated with systemic corticosteroids, which include gastric ulcers, weight gain, and psychological disturbances. Intravitreal corticosteroid injections, on the other hand, can trigger steroid-induced glaucoma, dramatically increase the rate of cataract development, and cause endophthalmitis [24]. In another 2016 study [25], the authors reported that two months after initiation of topical difluprednate, retinal vasculitis associated with birdshot chorioretinitis was resolved in both treated eyes of a patient as confirmed by fluorescein angiography. While difluprednate has demonstrated efficacy in inflammatory chorioretinopathies like VKH disease and birdshot chorioretinitis, these conditions differ fundamentally from CSR in that corticosteroids target their underlying inflammatory pathophysiology rather than potentially exacerbating choroidal dysfunction.

The predominant evidence from the literature suggests that an association exists between corticosteroids and the development of CSR [1, 6, 8-10]. Unless VKH is incorrectly diagnosed as CSR, difluprednate should be avoided for CSR treatment [26]. However, one study [27] based on a nationally representative sample of commercial insurance beneficiaries who received care between 2007 and 2015, reported that nearly 39% of 3418 CSR patients were prescribed systemic steroids at some point during the analysis period. Although CSR patients were significantly less likely to receive steroids within six months post-diagnosis compared with non-CSR patients (odds ratio, 0.72; 95% confidence interval, 0.59-0.89), the authors found that they were significantly more likely to receive steroids by two years post-diagnosis, with similar prescribing patterns for patients diagnosed by an ophthalmologist versus optometrist [27]. The authors did not examine the impact of steroid treatment on CSR patients. 

Hypotheses with interpretations

Barring a misdiagnosis, the continued use of steroids to reduce subretinal fluid may be dictated by several hypotheses. These proposed mechanisms remain theoretical and hypothesis-generating, as there is currently no direct clinical evidence demonstrating the therapeutic benefit of difluprednate in classic CSR.

One hypothesis is that CSR is a common endpoint for multiple mechanistic changes to the choriocapillaris: for some patients, this includes changes to the choriocapillaris that are exacerbated by corticosteroid use, while for others, the CSR is driven by inflammation that is improved by corticosteroids. Pothof et al. [12] found that topical prednisolone reduced intraretinal fluid in PPS patients and suggested that the etiology may be inflammatory or that the increase in IOP may tamponade leakage of the choriocapillaris. For the specific patient, it is imperative to record any appreciable change in IOP following a difluprednate treatment regimen. More recently, a retrospective case series [13] found that chronic pachychoroid disease spectrum (PPS and CSR fall under this umbrella term) can be effectively treated with a combination of topical difluprednate and oral loratadine to reduce retinal edema and subfoveal choroidal thickness. The authors hypothesized that the effectiveness of the therapy could potentially be linked to the regulation of mast cell degranulation, which necessitates a well-powered prospective randomized clinical trial.

Another hypothesis concerns the route of steroid administration that is crucial to the development of CSR. In this context, Bosquet et al. [28] described how glucocorticoids may lead to an overactivation of the mineralocorticoid pathway, thereby aggravating CSR. As the topical difluprednate diffuses into the eye, its penetration into further posterior tissues must decrease and likely falls dramatically once it interacts with the highly vascularized choriocapillaris. This pattern of absorption, or differential distribution of mineralocorticoid versus glucocorticoid receptors, may explain the differential effect that steroids have when administered systemically (therefore affecting the choroid and choriocapillaris concurrently) versus topically or intraocularly, as there are fewer reports of CSR being associated with the latter [1].

A third hypothesis is that the specific molecule difluprednate, as opposed to other topical corticosteroids, [6, 8-11] is helpful in the resolution of CSR. Compared to other ophthalmic corticosteroids, difluprednate has improved penetration, better bioavailability, rapid local metabolism, and stronger efficacy with a lower incidence of adverse effects [27, 28]. It is more potent and has superior dose uniformity than prednisolone due to its increased corneal penetration and being formulated as an emulsion, respectively [27-30]. Difluprednate is also more likely to increase IOP [30, 31]. However, there is evidence that suggests raising the IOP decreases SRF absorption time moderately and lowering the IOP increases it [32]. These effects were noted to be greater when the RPE had been damaged, and thus, the increased IOP from difluprednate may contribute to subretinal fluid absorption in the setting of CSR [32].

Quality assessment of studies

This review did not identify any published randomized clinical trial, which is the gold standard study design. One study recognized its retrospective design, the lack of a control group, and the short follow-up time as its limitations. A single patient case report does not allow the estimation of an effect size and would only provide descriptive or narrative results. A case series of more than one patient may allow narrative or quantitative synthesis only. Most other studies satisfied the population, intervention, comparator, outcome, and setting (PICOS) criteria. These studies generally demonstrated strong cohort selection methods, appropriate ascertainment of exposure and outcomes, and realistic follow-up time. 

Discussion

A significant proportion of the available corticosteroid-related literature involves immune-mediated inflammatory chorioretinal disorders, including uveitis, Vogt-Koyanagi-Harada disease, and birdshot chorioretinitis. These conditions differ fundamentally from classic CSR in their primary pathophysiology, as they are characterized by immune-driven inflammation rather than isolated choroidal hyperpermeability within the pachychoroid spectrum. Accordingly, mechanistic insights or therapeutic responses observed in inflammatory disorders cannot be directly extrapolated to classic CSR and are cited here primarily to inform potential receptor-level and pharmacologic considerations rather than to imply clinical equivalence. Difluprednate possesses distinct pharmacologic properties, including high glucocorticoid receptor affinity and enhanced posterior segment penetration, which may theoretically influence choroidal and RPE physiology. However, current evidence specific to CSR is limited to case reports, small retrospective series, and indirect mechanistic extrapolations from related but pathophysiologically distinct conditions. These data are hypothesis-generating and insufficient to establish efficacy or safety in this population.

Formal statistical summary methods, such as meta-analysis or meta-regression, were not attempted for this review because of the heterogeneity of included studies, the scarcity of directly relevant studies evaluating difluprednate for CSR, and the absence of any inferential analyses based on available quantitative information.

As discussed, difluprednate may have positive effects in certain cases, depending on the existence of pain and inflammation, as well as the comorbidities of the patients, including diabetes, stress, and hypertension; nevertheless, there is a notable gap in research regarding published clinical trials that specifically evaluate difluprednate in traditional CSR. The absence of this direct evidence is a significant finding of this review. Though this review did not find any prior studies that documented the impact of difluprednate eye drops for CSR treatment, the Google search identified two studies [33, 34], both from the Netherlands, on the use of topical steroids for the treatment of CSR, the characteristics of which are reported in Table 1. The retrospective cohort study [33] reported that the topical prednisolone acetate eyedrop resulted in a decrease in macular volume in 68% of the eyes affected by CSR and a complete resolution of foveal intra-or subretinal fluid in 22% of the eyes without noting any change in visual acuity. The currently ongoing registered clinical trial is conducting a pilot study with a randomized, single-blinded, placebo-controlled trial design to assess the safety and efficacy of steroid eye drops in patients suffering from CSR, using clinical, multimodal imaging, anatomical, and functional outcomes [34]. Its initial study protocol announced the recruitment of 40 participants, 18 years or older, who experienced visual problems accompanied by specific types of fluid visible in their eyes for three months or longer and would be randomly assigned to receive either a steroid eye drop or a placebo. The goal of this clinical trial is to advance understanding of potential new treatments for this eye condition. Notably, it did not specify the name of the topical steroid to be used, and the preliminary results have not been published yet. This ongoing clinical trial will need a statistical review when trial results are incorporated into future publications.

**Table 1:** Details of studies on the use of topical steroids for the treatment of CSR CSR - central serous chorioretinopathy

Author; setting	Study design data	Patients	Topical steroid use (dosage)	Outcomes
van den Tillaart et al., 2024 [33] Radboud University Medical Center (Netherlands)	Retrospective (data collected from medical records of patients with retinal disease confined to PDS between September 2020 and February 2023)	48 with 44 eyes with CSR	Prednisolone acetate 1% Eye Drops three-times daily	Decrease in macular volume in 30 eyes (68%). Foveal intraretinal or subretinal fluid resolved completely in 22% of the eyes. No change in visual acuity. Increase in intraocular pressure to 25 mmHg or higher in 16% of the eyes.
Yzer (Principal Investigator) et al., 2022 [34] Ongoing trial at Radboud University Medical Center and Rotterdam Eye Hospital (Netherlands)	Randomized single-blind placebo-controlled trial (registered in 2022)	40 patients aged 18 years or older with complex or severe CSR (pilot study registered in 2022)	Not specified three-times daily for four consecutive weeks	Change in subretinal and intraretinal fluid, visual acuity, and intraocular pressure.

## Conclusions

The enhanced potency of difluprednate does not inherently imply therapeutic benefit. Increased receptor activation may amplify either protective anti-inflammatory pathways or pathogenic mineralocorticoid receptor-mediated permeability mechanisms. Therefore, the clinical implications of difluprednate’s pharmacologic profile remain uncertain. The intention of this narrative literature review was not to position corticosteroids as broadly therapeutic in CSR, but rather to explore whether steroid signaling in pachychoroid diseases may be context-dependent and potentially heterogeneous across phenotypes. Specifically, the aim was to examine whether in select presentations or subtypes, localized inflammatory mechanisms might coexist with or modify the classical cortisol-driven model of disease. Several proposed mechanisms (e.g., mast cell modulation, intraocular pressure-mediated fluid absorption, and differential posterior steroid penetration) remain theoretical and unsupported by direct clinical evidence in CSR. These concepts should be explicitly identified as hypothesis-generating and should not be used to imply therapeutic efficacy.

The complex pathology of CSR is not well understood. Also, the differentiation between other ocular diseases with serous subretinal fluid and CSR can be challenging. Difluprednate could be prescribed for symptoms that could masquerade as CSR. At present, there is no high-quality evidence supporting routine clinical use of difluprednate that could also provide practical guidance regarding patient selection, dosing regimens, duration of therapy, monitoring strategies (including intraocular pressure), or expected timelines for anatomical or functional response. Currently available information underscores the need for prospective controlled studies to clarify whether corticosteroid signaling in CSR represents a therapeutic opportunity, a context-dependent phenomenon, or a potential source of harm. Until the results from such studies are published, it is premature and inadvisable to support routine clinical use of difluprednate outside research settings, particularly because its use as a form of therapy is contraindicated by the available evidence. Enhanced communication and collaboration among providers are required to ensure that clinical practice aligns with evidence-based recommendations.
